# The 4KEEPS study: identifying predictors of sustainment of multiple practices fiscally mandated in children’s mental health services

**DOI:** 10.1186/s13012-016-0388-4

**Published:** 2016-03-09

**Authors:** Anna S. Lau, Lauren Brookman-Frazee

**Affiliations:** 1 University of California, Los Angeles, USA; 2 University of California, San Diego, USA

**Keywords:** Children’s mental health, Fiscally driven implementation

## Abstract

**Background:**

Research to date has largely focused on predictors of adoption and initial implementation of evidence-based practices (EBPs), yet sustained implementation is crucial to deliver a return on investments in dissemination. Furthermore, most studies focus on single EBPs, limiting opportunities to study the fit between practice characteristics EBPs and implementation contexts.

**Methods/design:**

This observational study will characterize implementation sustainment and identify organizational and therapist characteristics that predict sustainment of multiple practices being implemented within a fiscal mandate in the largest public mental health system in the USA. Specific aims are to (1) characterize sustainment outcomes (volume/penetration, EBP concordant care); (2) use mixed methods to characterize inner context (agency- and therapist-level) factors and early implementation conditions; and (3) identify inner context factors and early implementation conditions that predict sustainment outcomes. This study will undertake original data collection and analysis of existing data sources to achieve its aims. Archived reports and documents will be used to characterize early implementation conditions in 102 agencies. Administrative claims data will be used to characterize volume and penetration outcomes over 8 years. Therapist and program manager surveys will be administered to characterize sustained EBP concordant care and inner context determinants of sustainment. An in-depth study in a subset of agencies will yield interview data and recordings of treatment sessions for validation of the EBP concordant care scale.

**Discussion:**

This project will yield new understanding of whether and how multiple EBPs can be sustained in public mental health systems undergoing a policy-driven community implementation effort. We will produce generalizable models for characterizing sustainment, including feasible and flexible measurement of practice across multiple EBPs. The findings will inform the development of implementation interventions to promote sustained delivery of EBPs to maximize their public health impact.

**Electronic supplementary material:**

The online version of this article (doi:10.1186/s13012-016-0388-4) contains supplementary material, which is available to authorized users.

## Background

Documented quality gaps between usual care (UC) and evidence-based practices (EBPs) [1, 2] have prompted large-scale implementation efforts in mental health (MH) systems [3–7]. Mandating EBPs in public managed care in children’s MH services began with the reform of services in Hawaii’s Department of Health following a 1999 consent decree [8]. By 2008, 90 % of state MH authorities reported strategies to install EBPs; 12 states had mandated the use of EBPs in public MH systems, with 8 states promoting, supporting, or requiring specific practices statewide [9]. These costly efforts provide natural laboratories to identify determinants of the sustainment of EBPs, an understudied topic in implementation science [10]. The Knowledge Exchange on Evidence-based Practice Sustainment (4KEEPS) study (R01 MH100134; MPIs Lau and Brookman-Frazee) is designed to understand the extent to which such investments result in sustained reach and use of multiple EBPs and identify determinants of sustainment that can be leveraged in novel implementation interventions. With few exceptions, most studies have focused on implementation outcomes of a single intervention. Yet MH systems are unlikely to implement a single intervention as patient needs vary [3, 11, 12]. Little is known about how sustainment outcomes and determinants of these outcomes vary by practice characteristics.

## Context of current study (see Additional file 1: History of Developments leading to the LACDMH PEI Implementation)

The Los Angeles County Department of Mental Health (LACDMH) is the nation’s largest county MH department, serving, on average, more than 250,000 county residents of all ages every year [13]. In 2010, LACDMH launched the Prevention and Early Intervention transformation with training and implementation support for an initial set of six evidence-based/informed practices (hereafter referred to as practices) to address a range of prevalent youth MH problems, including Cognitive Behavioral Interventions for Trauma in Schools (CBITS), Child-Parent Psychotherapy (CPP), Managing and Adapting Practices (MAP), Seeking Safety (SS), Trauma-Focused Cognitive Behavior Therapy (TF-CBT), and Triple P Positive Parenting Program (Triple P). Therapist trainings commenced in May, 2010, and in fiscal year 2010–2011, over 32,000 children and transition age youth were served in Prevention and Early Intervention (PEI) programs [14]. The timing of the current study relative to the maturity of PEI transformation permits examination of practice sustainment up to 8 years after adoption.

**Fig. 1:**
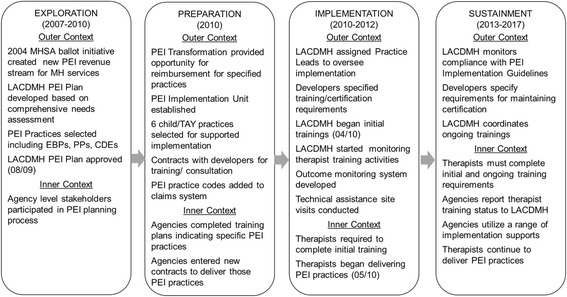
Applying Aarons et al.’s [15] EPIS Framework to the LACDMH Prevention and Early Intervention (PEI) timeline

We used the Exploration, Preparation, Implementation, and Sustainment (EPIS) framework [15] to frame the PEI transformation timeline and variables examined in the current study (see online resources Fig. 1). The EPIS model outlines four phases of implementation and highlights potential predictors of outcomes in the outer (e.g., funding, policy, organizational networks) and inner (e.g., organizational, leadership, therapist characteristics) contexts. Given the nature of the PEI transformation, “outer context” variables are shared across agencies and therapists, permitting us to isolate key aspects of the inner context (organizational and therapist variables) central in socio-technical models of implementation [16].

## Focus on sustainment

Sustainment is defined as the extent to which a newly implemented practice is maintained or institutionalized within a service setting’s ongoing, stable operations [17]. Stirman et al. [18] reviewed 125 studies examining sustainment across service settings and interventions and highlighted a general lack of methodological rigor. Recommendations included developing measures of multiple sustainment outcomes, assessing sustainment over years rather than at a single time point, and developing methods to measure intervention adaptation that may occur to achieve sustainment. Thus, 4KEEPS fills important gaps in the literature through long-term post-adoption follow-up, characterizing and understanding the impact of naturalistic adaptations of practices, and examining multiple indicators of sustainment of multiple practices within a “marketplace” of implementation.

It is essential to consider multiple sustainment outcomes jointly. One such sustainment outcome is penetration, defined as the integration of a practice within a service setting. It can be measured as the number of eligible individuals who use/deliver a treatment, divided by the number of eligible individuals [17]. An important limitation of indexing a penetration ratio alone is that it does not track the absolute scale of an implementation effort over time. Moreover, the denominators used to calculate penetration rates vary over time with changes in the workforce and system policies. Therefore, in addition to penetration, we assess volume of practices delivered over time in raw units of numbers of agencies, therapists, children, and units of service to characterize the scale of implementation impact.

Use of system administrative data provides one method of measuring penetration and volume and aligns with calls for use of these data to understand practice patterns and inform implementation efforts [19, 20]. However, by itself, penetration/volume of a practice within a workforce is insufficient to determine the maintained success of the implementation. Continued delivery with poor integrity to the practice model is unlikely to produce the expected value of the practice [21]. Likewise, sustained practice integrity has little impact if few therapists persist in implementation. Thus, indicators of practice penetration/volume and practice integrity are essential in the study of sustainment.

## Inner context determinants of sustainment outcomes

Within the PEI transformation, outer contextual factors are held relatively constant across agencies, as the fiscal mandate, contract conditions, reimbursement policies, and revenue stream apply uniformly to all agencies. As such, we will focus on identifying determinants of sustainment within the inner context.

### Organizational factors

Over 100 agencies are contracted with LACDMH to deliver at least one of the six practices of interest. These agencies vary widely in size, structure, and resources to support implementation which creates a unique opportunity to examine agency-level determinants of practice sustainment. Although research indicates that organization and therapist-level factors influence attitudes toward and adoption of EBPs [22–24], little research links these factors to sustainment. Organizational support for practices predicts adoption and initial implementation [23, 25], and organizational climate is associated with provider willingness to deliver practices [24, 26]. Specifically, leadership support, quality assurance structures, and staff incentives [27] predict positive therapist attitudes [28, 29] as well as workforce outcomes including lower turnover [23, 30]. Beyond early implementation phases, 4KEEPS will apply mixed methods to examine these associations with long-term sustained implementation of multiple practices at the therapist and agency levels.

### Therapist factors

Since 2010, approximately 8500 therapists have delivered at least one of the six practices of interest. Therapist background factors have been shown to influence both attitudes toward EBP [22], and therapist attitudes have been linked to practice behavior [31]. A survey conducted soon after the PEI transformation revealed that significant variance in therapist attitudes were explained by practice type and that practice-specific ratings of intervention appeal predicted concurrent therapist reports of EBP use [32]. 4KEEPS will prospectively examine relations between dimensions of therapist attitudes and sustained use of practices over time as indexed by administrative, self-report, and observational data.

There is growing attention to the role of therapist adaptations to practices and their implications for implementation outcomes [18, 33, 34]. Although it is often implicitly expected that practices be delivered with efficacy trial adherence standards, this is at odds with contingencies in UC settings [35]. Therapists commonly raise the concern that practices may not fit the needs of clients seen in UC and must be adapted, particularly for ethnic minority groups not represented in controlled trials [36–39]. Therapists may adapt by reframing interventions, incorporating techniques or components to promote acceptance or address group-specific needs [40]. Such investments in tailoring practices may increase commitment to delivering the EBP long term [41, 42]. On the other hand, inappropriate modifications or omissions represent drift [34]. It is unclear the extent to which community therapists engage in adaptations and whether they are consistent or inconsistent with practice integrity.

The overall objectives of 4KEEPS project are to characterize sustainment outcomes and identify inner context factors that predict the sustained penetration and use of multiple practices delivered through a fiscal mandate in children’s MH services. Supplemental aims address client-level determinants of implementation and potential racial disparities (see Additional file 2: 4KEEPS Disparities Supplement Aims).

#### Aim One: Characterize sustainment outcomes of six practices within the PEI transformation

##### EBP concordant care

Fidelity refers to the degree to which an intervention is delivered as intended by the program developers [43]. Fidelity to EBP is considered a central implementation outcome [17]. However, traditional fidelity measures may not be appropriate in the current study context. Assessments of therapist practice must be both effective and efficient to track the success of implementation efforts large enough to impact public health [21, 44, 45]*.* Direct observation, the gold standard in evaluating fidelity in controlled trials, is not feasible in large-scale UC implementation. Moreover, measuring fidelity across interventions that vary in content and focus presents a challenge when multiple EBPs are adopted.

Therefore, we assess a related construct to characterize sustained use of a practice—EBP concordant care. We depart from protocol-specific fidelity measures that include features specific to manualized protocols that do not necessarily generalize across interventions within a family of treatments shown to be effective for ameliorating a MH condition. EBP concordant care indexes the degree to which a therapist’s practice resembles the essential strategies one would expect within an evidence-based protocol for a given problem focus. Drawing from established tools used to characterize UC therapist practice, we developed the *EBP Concordant Care Assessment (ECCA).* The ECCA will provide a common metric to assess the extent to which a therapist delivers practices considered “essential” in EBPs for six major child MH targets: anxiety, depression, conduct problems, trauma, attachment problems, and substance use. This measure is completed by therapists and will be validated against observational coding.

#### Practice volume and penetration

Administrative claims data from LACDMH will be used to measure the volume and penetration of the six practices over time using a variety of units of analysis from FY10-11 through FY17-18. The volume of any given practice is defined as the numbers of agencies continuing to be reimbursed for the practice, therapists continuing to claim to the practice, unique clients served by the practice, and units of services being provided within each practice. Within PEI, providers must submit claims to be reimbursed for any service rendered and each claim must specify the practice. Penetration is calculated as the volume (claims, clients, therapists, agencies) for a given practice divided by the total volume across practices. Claims data will index volume and penetration for 8 years from the outset of the PEI transformation.

#### Aim two: Use mixed methods to characterize inner context factors and early implementation conditions that potentially predict EBP sustainment

The ultimate goal of this study is to identify factors associated with the long-term sustainment of multiple practices introduced in a major system reform toward EBP implementation. Challenges with this goal include the potentially time-varying nature of inner context factors and the time period of the study (i.e., assessing these factors after initial implementation). Thus, we use a multi-method approach including repeated measures of potentially time-varying inner context factors and analyses of archival data on early implementation conditions. We apply a mixed quantitative and qualitative approach since their combined use provides a better understanding of the implementation context than either approach alone [46]. Qualitative methods (interviews and archival document review) are used to obtain in-depth understanding of the conditions associated with success in sustainment while quantitative methods (survey methods) test hypotheses based on existing models of implementation. The key constructs of interest include agency-level organizational factors (Agency Structure, Climate, Leadership, and Early Implementation Conditions) and therapist-level attitudes toward and adaptations to practices.

#### Aim Three: Identify inner context and early implementation conditions that determine sustainment outcomes

We will use multi-level modeling to examine predictors of volume/penetration trajectories and end point estimates of therapist delivery of EBP concordant care. We will examine whether individual determinants interact to impact sustainment outcomes and whether determinants differ based on the type of sustainment outcome (i.e., EBP concordant care vs. volume/penetration).

## Methods/design

### Existing data sources

#### Claims data

Administrative claims data will index PEI-reimbursed claims for the six practices from the outset of the reform in FY 2009–2010 through the end of the study in FY 2018–2019. Data obtained from 2009–2014 (19 fiscal quarters) have yielded over 2.3 million psychotherapy claims for more than 87,000 unique child and transition age youth (TAY) clients provided by more than 8500 MH clinicians within 94 agencies (Brookman-Frazee et al., under review). Gross volume and penetration will be calculated at each level (claims, client, therapist, agency) per fiscal quarter for each practice. Claims data for each practice may have up to a four-level structure with claims nested within fiscal quarter, nested within therapists, within agencies. Because some therapists will transition across agencies within LACDMH over time, cross-classified models will be use to account for multiple membership nesting [47]. Claims will also be indexed at the therapist level to characterize the volume of claims per therapist for each practice relative to their total claims across practices.

### Document extraction

Since the study commenced years after the PEI transformation launched, prospective assessment of early implementation conditions was not possible. However, readiness for implementation and early implementation integrity are two factors that may predict our sustainment outcomes of interest. As such, we will extract information from detailed documentation generated by Technical Assistance Site Visits that were carried out by the LACDMH from June 2012 through December 2013. The purpose of the visits was to support agencies in claiming procedures and to assess implementation milestones (e.g., therapist training, instantiation of supervision/consultation/fidelity monitoring, outcome tracking). Agencies completed a Pre-Site Visit Questionnaire (PSVQ) prior to site visits which included a 3-h meeting led by LACDMH staff and agency program managers, with supervisors, and therapists. Site Visit Reports (SVRs) summarizing discussions were produced within 3 weeks of each visit. The PSVQs and SVRs will be coded to provide a measure of early implementation conditions at the agency level.

### Original data collection

#### Surveys

Survey data will be collected from therapists and program managers across agencies contracted to provide at least one of the six practices of interest to children or transition age youth. Therapists currently billing for psychotherapy services for at least one of them are eligible. We will enumerate our therapist sample by contacting program managers at each contracted agency to request email contacts for all eligible therapists. Participants will be invited to participate with a personalized link to the online survey via email. The sampling frame for the survey in fiscal year 2013–2014 included 102 agencies; we anticipate an agency-level response rate of 75 %, yielding a sample of 75 agencies. We anticipate an average of 18–24 eligible therapists per agency and a response rate of 50 % at the therapist level, to yield a survey sample of approximately 800 therapists.

Table 1 (see online resources) lists survey measures of sustainment outcomes and agency- and therapist-level predictors of sustainment to be included in therapist and program manager surveys. All established measures have strong evidence of reliability and validity. Two surveys will be fielded over the course of the study in years 2 and 4 to provide repeated measures of the inner context determinants of sustainment (organizational and therapist characteristics). These data will permit examination of the stability of inner context factors and will provide a more temporally proximal prediction of sustainment outcomes during the final year of the study.

**Table 1:** 4KEEPS key constructs and measures as predictors of sustainment outcomes

Measure	Subscales	Sample items	Citations	Th	PM
Therapist attitudes					
Perceived Characteristics of Intervention Scale (PCIS)	Relative advantage, compatibility, complexity, potential for reinvention	(Practice) is more effective than other therapies I have used.	Cook JM, Thompson R, Schnurr PP. Perceived characteristics of intervention scale: development and psychometric properties. Assessment. 2015;22(6):704–14. doi:10.1177/1073191114561254.	x	x
Evidence-Based Practice Attitude Scale (EBPAS)	Divergence, openness, limitations (practice-specific)	I like to use new types of therapy/interventions to help my clients.	Aarons GA, Glisson C, Hoagwood K, Kelleher K, Landsverk J, Cafri G, et al. Psychometric properties and U.S. national norms of the Evidence-Based Practice Attitude Scale (EBPAS). Psychol Assess. 2010;22(2):356–65*.* doi:10.1037/a0019188.	x	
		(Practice) detracts from truly connecting with my clients.			
Knowledge and confidence		I am well prepared to deliver (Practice) even with challenging clients.	Project developed	x	
		I am confident in my ability to implement (Practice).			
Implementation support					
Practice-specific implementation support	–	Rate the frequency that the following are part of ongoing supervision for: a.Live observation b.Review of recorded session c.Case discussion d.Review of client progress monitoring (outcomes, dashboards) …	Project developed	x	x
Organizational context					
Organizational climate measure	Autonomy, Rational Goal (Performance Feedback), HR Involvement (Therapists)	Program managers and/or agency leaders let people make their own decisions much of the time.	Patterson MG, West MA, Shackleton VJ, Dawson JF, Lawthom R., Maitlis S, et al. Validating the organizational climate measure: links to managerial practices, productivity and innovation. J Organ Behav. 2005;26:379–408. doi:10.1002/job.312.	x	x
Organizational readiness to change	Staffing, Cohesion, Stress	Frequent therapist turnover is a problem for your program.	Lehman WEK, Greener JM, Simpson DD. Assessing organizational readiness for change. J Subst Abuse Treat. 2002;22:197–209. doi:10.1016/S0740-5472(02)00233-7		x
Maslach Burnout Inventory (adapted)	Exhaustion, Personal Accomplishment	I feel emotionally drained from my work.	Schaufeli WB, Leiter MP. Maslach Burnout Inventory–general survey. In: The Maslach burnout inventory-test manual. 1996. p. 19–26.	x	
		I feel I’m positively influencing the lives of my clients through my work.			
Therapist adaptations					
Adaptations of evidence-based practices	–	I modify how I present or discuss the components of (Practice).	Stirman SW, Miller CJ, Toder K, Calloway A. Development of a framework and coding system for modifications and adaptations of evidence-based interventions. Implement Sci. 2013;8(65):1–12. doi:10.1186/1748-5908-8-65.	x	
		I apply (Practice) to novel populations.			
Barriers to implementation (client-level)					
Emergent life event (ELE) and engagement measure	–	Did the client or client’s caregiver raise a stressful life event that was not the intended focus of session? If yes, to what extent were you able to carry out your intended activities or return to the focus of session?	Project developed	x	

### In-depth study

Multi-method data including surveys, qualitative interviews, and behavioral observations will be collected in a subset of agencies. The objectives of the in-depth component of the 4KEEPS study are twofold. First, it will furnish qualitative interviews essential to our mixed methods (QUAN + QUAL) design, with quantitative data and qualitative data given equal weight. Combined analyses will be used for convergence (i.e., triangulation of data across methods to determine if the same conclusion is reached) and complementarity (i.e., qualitative data provide depth of understanding while quantitative data provide breadth of understanding). Second, the in-depth study will allow us to validate therapist self-reports of EBP concordant care against behavioral observations.

The sampling frame for the in-depth study will be the agencies enumerated into the survey sample described above. We will target a sample of 120 therapists and at least one program manager at each agency providing administrative oversight for PEI practices. Therapist interview guides will focus on training experiences, attitudes toward practices, and adaptations made to practices. Program manager interview guides will focus on barriers and facilitators of implementation of practices, the impact of adopting practices, and, when relevant, reasons for de-adoption of practices. Furthermore, therapists will provide self-reports of practice implementation on the ECCA and supply audio recordings of three therapy sessions for three clients.

#### In-depth study measure development activities

##### ECCA development

Multiple inputs have informed the development of a generic measure of EBP concordant care in the treatment of six child MH problem areas: anxiety, attachment problems, conduct, depression, substance abuse, and trauma addressed by the six practices of interest. The ECCA will include a common set of items regardless of what practice the therapist indicates they are using, with separate summary scores for each family of interventions. Item content was adapted from existing practice inventories [48–51], and additional items were derived from intervention materials for practices not reflected in existing inventories. Practice experts, including intervention developers (i.e. authors of treatment manuals), and master trainers were enlisted in the item development process and in the determination of EBP concordance for the treatment of child MH target areas. For validation, we will examine the correspondence between therapist self-report on the ECCA and observer ratings at the session level using a parallel ECCA observational coding system.

##### Therapist adaptations of practices

To develop a measure of therapist adaptations to practices, we will use mixed methods sequentially (QUAN → qual → QUAN). In the first therapist survey (year 2), we will collect quantitative data to characterize therapist adaptations on a large scale using a questionnaire adapted from the Stirman et al. [52, 53] framework of adaptations and modifications of EBPs. Qualitative data from therapist interviews will later inform the refinement of the second therapist survey (year 4), using a Rapid Assessment Procedure (RAP) designed to provide depth and specificity in quantitative measures for assessing observed phenomena [54].

## Discussion

The 4KEEPS project will yield new understanding of whether and how multiple EBPs can be sustained in public MH systems undergoing policy-driven reform. This project is significant for many reasons. First, conducting an observational study of UC implementation of this magnitude maximizes the representativeness of agencies and therapists and the resultant generalizability of findings. Most studies have focused on investigator-driven implementation, whereas PEI was driven by system-level stakeholders within a major policy reform. Unlike experimental studies, observational studies of system change are less likely to include only the ideal organizations with the greatest motivation and readiness to implement. There is greater independence from EBP developers and their implementation “superstructures” when studying implementation as usual [55]. The outer context policy change is likely to be a model for EBP implementation in public MH systems. Yet we know little about how such policies may unfold in the years following adoption.

Second, examining the sustainment of multiple practices is essential because large-scale EBP reform is not likely to involve dissemination of single interventions because any single EBP will not address all client MH needs. Chorpita et al. [12] found that a minimum of eight EBPs would be required to cover the range of problems child MH problems represented in LACDMH. The PEI transformation provides an opportunity to study the determinants of sustainment across multiple EBPs in a large, organizationally diverse, and ethnically diverse service system. In this context, penetration of a practice is relative to other available practices. This shifts the focus away from questions about whether a given intervention is likely to be sustained toward identifying characteristics of practices and their fit with organizations that promote sustainment within a marketplace of dissemination.

Third, methodological features of the study are important for the advancement of sustainment research. Metrics of practice volume and penetration and EBP concordant are necessary to assess the public health impact promised by dissemination of EBPs. Using generalizable methods, the study will produce feasible metrics for assessing therapist adaptations and integrity of EBP implementation across practices. Ultimately, the findings will inform the development of implementation interventions to promote sustained delivery of EBPs to maximize their public health impact.

## Additional files

Additional file 1: History of Outer Context Developments leading to the LACDMH PEI Implementation. (DOCX 13 kb)

Additional file 2: 4KEEPS Disparities Supplement Aims. (DOCX 33 kb)

## Abbreviations

4KEEPS: Knowledge Exchange on Evidence-based Practice Sustainment
EBP: evidence-based practice
LACDMH: Los Angeles County Department of Mental Health
MHSA: Mental Health Services Act
PEI: Prevention and Early Intervention
ECCA: Evidence-based Practice (EBP) Concordant Care Assessment
CBITS: Cognitive Behavioral Interventions for Trauma in Schools
CPP: Child-Parent Psychotherapy
MAP: Managing and Adapting Practices
SS: Seeking Safety
TF-CBT: Trauma-Focused Cognitive Behavior Therapy
Triple P: Triple P Positive Parenting Program
