# Supplementary material for: The 4KEEPS study: identifying predictors of sustainment of multiple practices fiscally mandated in children’s mental health services
Source: Implement Sci. 2016 Mar 9;11:31. doi: 10.1186/s13012-016-0388-4 (PMC4784305; doi:10.1186/s13012-016-0388-4)
Supplement: Supplementary file 1 — History of Outer Context Developments leading to the LACDMH PEI Implementation. (DOCX 13 kb) [file 13012_2016_388_MOESM1_ESM.docx]

**History of Outer Context Developments leading to the LACDMH PEI Implementation**

The opportunity for this observational study of the implementation of multiple practices in children’s mental health emerged following a series of state-level developments in California. The Mental Health Services Act (MHSA) was enacted from a state ballot measure initiative passed in November, 2004 that assessed a 1% tax on the income of individuals who make in excess of $1 million per year. The funding generated by this tax was earmarked for specific types of mental health services. This revenue stream resulted in changes in workforce training, systems of care for clients across the age spectrum and an increased focus on delivery of effective practices with measurable outcomes statewide. The Los Angeles County Department of Mental Health (LACDMH) is the nation’s largest county MH department, serving, on average, more than 250,000 County residents of all ages every year [[1](#_ENREF_1)]. In July, 2010, LACDMH elected to utilize MHSA Prevention and Early Intervention (PEI) funding to serve as a catalyst to utilize Evidence-Based, Promising and Community Defined Practices to intervene earlier in the course of mental illness. In response to external factors including public policies and initiatives that mandated behavioral health reform in the context of an overall state budget shortfall, LACDMH offered agencies the opportunity to receive reimbursement through PEI for the delivery of 52 practices approved in the county’s PEI Plan to achieve the goals of prevention and early intervention. Consequently, the contracts for agencies that served children and transition-age youth were amended to include PEI funds and implementation guidelines. Agency leaders selected from among the 52 practices based on their perceived needs and preferences.

References

1. Los Angeles County Department of Mental Health. About DMH. <http://dmh.lacounty.gov/wps/portal/dmh/aboutdmh>. Accessed 29 Jan 2016.
