# Supplementary material for: The 4KEEPS study: identifying predictors of sustainment of multiple practices fiscally mandated in children’s mental health services
Source: Implement Sci. 2016 Mar 9;11:31. doi: 10.1186/s13012-016-0388-4 (PMC4784305; doi:10.1186/s13012-016-0388-4)
Supplement: Supplementary file 2 — 4KEEPS Disparities Supplement Aims. (DOCX 33 kb) [file 13012_2016_388_MOESM2_ESM.docx]

**4KEEPS Disparities Supplement Aims**

We received an administrative supplement to the parent study (R01 MH100134-S) to examine the extent to which there may be racial disparities in implementation outcomes in the PEI Transformation. Our aims are to examine potential barriers to the implementation of EBPs that may co-vary with client race/ethnicity and may drive disparities in EBP concordant care. As such, we expand our predictors of sustainment outcomes to include client- and session-level predictors of EBP concordant care.

Over 87% of families served in PEI services in FY 2010-11 were ethnic minorities, and all are low-income Medicaid-eligible families. Disparities in access to children’s MH services are well documented (e.g., [[1](#_ENREF_1), [2](#_ENREF_2)]). Furthermore, ethnic minority children appear to receive lower quality care than non-Hispanic White youth for MH conditions including autism and depression [[3](#_ENREF_3), [4](#_ENREF_4)]. However, few studies have examined potential racial/ethnic disparities in EBP implementation. The implementation of EBPs may reduce disparities in care [[5](#_ENREF_5)], however, implementation may be challenging in the context of familial stressors and cultural barriers faced by ethnic minority families in public sector services.

Disadvantaged minorities are more likely than non-Hispanic Whites to face stress involving violence exposure, discrimination, poverty, family separation, inadequate housing and education, and poor health [[6-11](#_ENREF_6)]. Emergent Life Events (ELEs [[12](#_ENREF_12)]) are acute stressors disclosed by the client or caregiver unexpectedly during treatment that were not the planned focus of the treatment session. ELEs are common among families receiving LACDMH services and therapist reports of ELE occurrence are associated with disrupted implementation of EBPs.

Furthermore, EBPs may not fit the needs of ethnic minority families seen in usual care settings who are inadequately represented in controlled trials [[13](#_ENREF_13), [14](#_ENREF_14)]. The most common therapist complaint about EBPs concerns a perceived lack of flexibility to tailor treatment to client characteristics, preferences, and/or culture [[15](#_ENREF_15)]. Hispanic therapists cite these concerns more often than non-Hispanic White therapists [[16](#_ENREF_16)]. Implementing EBPs with ethnic minority families may be complicated by barriers related to: (1) acceptability of EBPs; and (2) learning culturally unfamiliar skills taught in EBPs [[17](#_ENREF_17), [18](#_ENREF_18)]. Therapist adaptations may improve family engagement without compromising fidelity, however, inappropriate modifications may threaten practice integrity [[19-21](#_ENREF_19)]. The extent to which therapists perceive or encounter cultural barriers in delivering EBPs to minority groups might explain disparities in implementation.

To achieve these goals within the in-depth component of the study we will work with therapists to oversample groups less well represented in LACDMH (e.g., non-Hispanic White, African-American, Asian-American/Pacific Islander). For each client sampled, therapists will provide ECCA data and recordings for 3 sessions for each client (n= 1080). In addition, to ECCA coding, session recordings and therapist self-reports will be used to characterize barriers to implementation at the session-level needed to examine mechanisms underlying potential disparities in practice implementation.

With the support of this administrative supplement to address disparities in practice sustainment, we will explore the following research questions: Are there disparities in sustained use and delivery of EBP concordant care as a function of client race/ethnicity? Secondly, are disparities in sustained implementation explained by increased therapist encounters with barriers to delivery of practices, including cultural barriers to learning or family ELEs?

References

1. Elster A, Jarosik J, VanGeest J, Fleming M. Racial and ethnic disparities in health care for adolescents: A systematic review of the literature. Arch Pediatr Adolesc Med. 2003;157(9):867–74. doi:10.1001/archpedi.157.9.867.

2. Kataoka SH, Zhang L, Wells KB. Unmet need for mental health care among U.S. children: Variation by ethnicity and insurance status. Am J Psychiatry. 2002;159(9):1548–55. doi:10.1176/appi.ajp.159.9.1548.

3. Alexandre PK, Martins SS, Richard P. Disparities in adequate mental health care for past-year major depressive episodes among Caucasian and Hispanic youths. Psychiatr Serv. 2009;60(10):1365–71. doi:10.1176/appi.ps.60.10.1365.

4. Magaña S, Parish SL, Rose RA, Timberlake M, Swaine JG. Racial and Ethnic Disparities in Quality of Health Care Among Children with Autism and Other Developmental Disabilities. Intellect Dev Disabil. 2012;50(4):287–99. doi:10.1352/1934-9556-50.4.287.

5. Davis TD, Deen T, Bryant-Bedell K, Tate V, Fortney J. Does minority racial-ethnic status moderate outcomes of collaborative care for depression? Psychiatr Serv. 2011;62(11):1282–8. doi:10.1176/appi.ps.62.11.1282.

6. Crouch JL, Hanson RF, Saunders BE, Kilpatrick DG, Resnick HS. Income, race/ethnicity, and exposure to violence in youth: Results from the national survey of adolescents. J Community Psychol. 2000;28(6):625–41. doi:10.1002/1520-6629(200011)28:6<625::AID-JCOP6>3.0.CO;2-R.

7. Gudiño OG, Nadeem E, Kataoka SH, Lau AS. Relative impact of violence exposure and immigrant stressors on Latino youth psychopathology. J Community Psychol. 2011;39(3):316–35. doi:10.1002/jcop.20435.

8. Hatch S, Dohrenwend B. Distribution of traumatic and other stressful life events by race/ethnicity, gender, SES and age: A review of the research. Am J Community Psychol. 2007;40(3-4):313–32. doi:10.1007/s10464-007-9134-z.

9. Jackson JS, Knight KM, Rafferty JA. Race and unhealthy behaviors: Chronic stress, the HPA axis, and physical and mental health disparities over the life course. Am J Public Health. 2010;100(5):933–9. doi:10.2105/ajph.2008.143446.

10. Turner R, Lloyd DA. Stress burden and the lifetime incidence of psychiatric disorder inyoung adults: Racial and ethnic contrasts. Arch Gen Psychiatry. 2004;61(5):481–8. doi:10.1001/archpsyc.61.5.481.

11. Turner RJ, Avison WR. Status variations in stress exposure: Implications for the interpretation of research on race, socioeconomic status, and gender. Journal of Health and Social Behavior. 2003;44(4):488–505. doi:10.2307/1519795.

12. Chorpita BF, Korathu-Larson P, Knowles LM, Guan K. Emergent life events and their impact on service delivery: Should we expect the unexpected? Prof Psychol Res Pr. 2014;45(5):387393. doi:10.1037/a0037746.

13. Aisenberg E. Evidence-based practice in mental health care to ethnic minority communities: Has its practice fallen short of its evidence? Soc Work. 2008;53(4):297–306. doi:10.1093/sw/53.4.297.

14. Bernal G, Scharró-del-Río MR. Are empirically supported treatments valid for ethnic minorities? Toward an alternative approach for treatment research. Cultur Divers Ethnic Minor Psychol. 2001;7(4):328–42. doi:10.1037/1099-9809.7.4.328.

15. DiMeo MA, Moore GK, Lichtenstein C. Relationship of evidence-based practice and treatments: A survey of community mental health providers. J Community Psychol. 2012;40(3):341–57. doi:10.1002/jcop.20516.

16. Aarons GA, Cafri G, Lugo L, Sawitzky A. Expanding the domains of attitudes towards evidence-based practice: The Evidence Based Practice Attitude Scale-50. Adm Policy Ment Health. 2010;39(5):331–40. doi:10.1007/s10488-010-0302-3.

17. Lau AS, Fung JJ, Ho LY, Liu LL, Gudino OG. Parent training with high-risk immigrant chinese families: A pilot group randomized trial yielding practice-based evidence. Behav Ther. 2011;42(3):413–26. doi:10.1016/j.beth.2010.11.001.

18. Lau AS, Fung JJ, Yung V. Group parent training with immigrant chinese families: Enhancing engagement and augmenting skills training. Journal of Clinical Psychology. 2010;66(8):880–94. doi:10.1002/jclp.20711.

19. Stirman SW, Gutner CA, Crits-Christoph P, Edmunds J, Evans AC, Beidas RS. Relationships between clinician-level attributes and fidelity-consistent and fidelity-inconsistent modifications to an evidence-based psychotherapy. Implement Sci. 2015;10:115. doi:10.1186/s13012-015-0308-z.

20. Lau AS. Making the case for selective and directed cultural adaptations of evidence-based treatments: Examples from parent training. Clin Psychol Sci Pr. 2006;13(4):295–310. doi:10.1111/j.1468-2850.2006.00042.x.

21. Cabassa LJ, Baumann AA. A two-way street: Bridging implementation science and cultural adaptations of mental health treatments. Implement Sci. 2013;8:90. doi:10.1186/1748-5908-8-90.
